# Comparison of adverse pregnancy and birth outcomes using archival medical records before and during the first wave of the COVID-19 pandemic in Kinshasa, Democratic Republic of Congo: a facility-based, retrospective cohort study

**DOI:** 10.1186/s12884-022-05291-w

**Published:** 2023-01-16

**Authors:** Patrick J. Arena, Camille Dzogang, Adva Gadoth, Dalau Mukadi Nkamba, Nicole A. Hoff, David Kampilu, Michael Beia, Hui-Lee Wong, Steven A. Anderson, Didine Kaba, Anne W. Rimoin

**Affiliations:** 1 Department of Epidemiology, Fielding School of Public Health, University of California, Los Angeles, Los Angeles, California, USA; 2 UCLA-DRC Health Research and Training Program, Kinshasa, Democratic Republic of Congo; 3 Kinshasa School of Public Health, University of Kinshasa, Kinshasa, Democratic Republic of Congo; 4 Office of Biostatistics and Pharmacovigilance, U.S. Food and Drug Administration, Silver Spring, Maryland, USA

**Keywords:** Adverse birth outcomes, GAIA, Democratic Republic of Congo, Medical records, COVID-19, Maternal immunization

## Abstract

**Background:**

Little research has been conducted on the impact of the coronavirus disease 2019 (COVID-19) pandemic on either birth outcomes or the ability of archival medical records to accurately capture these outcomes. Our study objective is thus to compare the prevalence of preterm birth, stillbirth, low birth weight (LBW), small for gestational age (SGA), congenital microcephaly, and neonatal bloodstream infection (NBSI) before and during the first wave of the COVID-19 pandemic in Kinshasa, Democratic Republic of Congo (DRC).

**Methods:**

We conducted a facility-based retrospective cohort study in which identified cases of birth outcomes were tabulated at initial screening and subcategorized according to level of diagnostic certainty using Global Alignment of Immunization Safety Assessment in pregnancy (GAIA) definitions. Documentation of any birth complications, delivery type, and maternal vaccination history were also evaluated. The prevalence of each birth outcome was compared in the pre-COVID-19 (i.e., July 2019 to February 2020) and intra-COVID-19 (i.e., March to August 2020) periods via two-sample *z*-test for equality of proportions.

**Results:**

In total, 14,300 birth records were abstracted. Adverse birth outcomes were identified among 22.0% and 14.3% of pregnancies in the pre-COVID-19 and intra-COVID-19 periods, respectively. For stillbirth, LBW, SGA, microcephaly, and NBSI, prevalence estimates were similar across study periods. However, the prevalence of preterm birth in the intra-COVID-19 period was significantly lower than that reported during the pre-COVID-19 period (8.6% vs. 11.5%, *p* < 0.0001). Furthermore, the level of diagnostic certainty declined slightly across all outcomes investigated from the pre-COVID-19 to the intra-COVID-19 period. Nonetheless, diagnostic certainty was especially low for certain outcomes (i.e., stillbirth and NBSI) regardless of period; still, other outcomes, such as preterm birth and LBW, had moderate to high levels of diagnostic certainty. Results were mostly consistent when the analysis was focused on the facilities designated for COVID-19 care.

**Conclusion:**

This study succeeded in providing prevalence estimates for key adverse birth outcomes using GAIA criteria during the COVID-19 pandemic in Kinshasa, DRC. Furthermore, our study adds crucial real-world data to the literature surrounding the impact of the COVID-19 pandemic on maternal and neonatal services and outcomes in Africa.

**Supplementary Information:**

The online version contains supplementary material available at 10.1186/s12884-022-05291-w.

## Introduction

Despite tremendous progress by low- and middle-income countries (LMICs) to reduce global child morbidity and mortality, infectious diseases remain a leading cause of death among children under five years of age [1]. Maternal immunization seeks to address this problem by reducing childhood morbidity and mortality associated with vaccine-preventable diseases through the transfer of protective antibodies from mother to child across the placental barrier [2–4]. In order to better facilitate research into the safety of maternal immunization approaches both within and across countries, the Global Alignment of Immunization Safety Assessment in pregnancy (GAIA) project created standardized guidelines and case definitions for the identification of maternal immunization and neonatal outcomes [5]. Previously, we evaluated the feasibility of facility-based archival medical records for the identification of neonatal health outcomes as well as maternal vaccination using GAIA case definitions in Kinshasa Province, Democratic Republic of Congo (DRC); we concluded that these archival medical records could feasibly be utilized to screen and identify a variety of pregnancy and birth outcomes, such as stillbirth, preterm birth, low birth weight (LBW), small for gestational age (SGA), and congenital microcephaly (despite some issues associated with interfacility variability) [6].

In that study, we assessed medical records from July 1, 2019 to February 28, 2020, i.e., immediately prior to the recognized onset of the coronavirus disease 2019 (COVID-19) pandemic in DRC. More specifically, the first case of COVID-19 in DRC was reported on March 10, 2020 [7]. Within two weeks of this first case, the Congolese government declared a nationwide state of public health emergency. In Kinshasa (i.e., the capital), the governor announced the total confinement of the commune of Gombe, an affluent area where many key governing bodies are stationed [8, 9]. Many feared that such measures could lead to decreased access to healthcare, particularly maternal and neonatal health services, as well as increased rates of adverse pregnancy and birth outcomes throughout DRC and Africa in general [10–13]. Nonetheless, recent research from eight sub-Saharan African countries (including DRC) has indicated that the most substantial reductions in medical services during the pandemic were reported for outpatient consultations and childhood vaccinations, while service utilization for reproductive and maternal healthcare was mixed [14]. Investigations in other LMICs (i.e., South Africa [15], Pakistan [16], and Nepal [17]) have suggested that lockdowns led to decreases in routine immunizations, institutional delivery rates, and/or the utilization of child healthcare services. However, a recent interrupted time series analysis found that maternal health services and vaccinations were not significantly affected by the pandemic itself or by lockdown measures during the first wave of the pandemic in Kinshasa [18]. Beyond policy implications though, the pandemic may negatively impact pregnant women and their newborns directly via infection with severe acute respiratory syndrome coronavirus 2 (SARS-CoV-2) and/or the negative consequences of stress associated with fear of the virus or economic implications of lockdowns [19–21].

In the context of this societal disruption and potential increase in healthcare system burden, it is vital to investigate the effect of the COVID-19 pandemic and associated policies on adverse birth outcomes and birth complications (which could be impacted by prenatal coronavirus exposures) as well as the pandemic’s effect on medical records-keeping accuracy, which may serve as an indicator of overall health system robustness. Therefore, we retrospectively surveilled and applied GAIA case definitions to maternal tetanus immunization and adverse birth outcomes during the first six months of the COVID-19 pandemic across delivery facilities throughout Kinshasa Province; we then compared these results to our previous findings from a similar investigation between July 1, 2019 to February 28, 2020 (i.e., the pre-COVID-19 period) [6]. Furthermore, we estimated the prevalence of adverse birth outcomes of interest, performed crude comparisons against these same measures in the pre-COVID-19 period, and described rates of maternal tetanus immunization. Lastly, we also compared birth complications and delivery type across the two time periods to provide a more comprehensive look at the impact of the pandemic on pregnancy and maternal care.

## Methods

### Study design and procedures

The overall study design, site selection, and data collection procedures have been described elsewhere [6]. Briefly, we conducted a facility-based retrospective cohort study that began data collection in January 2020. Archival medical records of mother–child pairs from births taking place in delivery centers throughout Kinshasa Province between July 1, 2019 and February 28, 2020 were first collected and digitized. Due to the COVID-19 pandemic, a second phase of data collection and digitization was performed on the archival medical records of mother–child pairs from births taking place in the same delivery centers between March 1, 2020 and August 31, 2020. The ten study sites, which were all within Kinshasa Province, in this study were as follows: Bomoi, Bondeko, Lisanga, Siloe Bdom, and Bosembo health centers; Esengo, Mokali, Saint Joseph, and Kinshasa general hospitals; and Ngaliema clinic. Data abstractors, who were trained in data collection methodology prior to initiation of study activities, collected and then digitally uploaded data from paper medical records stored at each study site in a two-stage process. First, each birth record was digitally recorded using Open Data Kit software and uploaded to an online server; then, these data were downloaded and screened to identify births that appeared to meet definitions for any of the six adverse birth and neonatal outcomes of interest, referred to here as “cases.” If a new birth was identified as a case, a supplementary module to collect additional information on the mother was implemented.

### Outcome measures

This study had three primary outcomes: adverse birth outcomes (including their GAIA classifications), maternal antenatal vaccination (including its GAIA classification), and birthing characteristics. The six adverse birth outcome endpoints included: neonatal bloodstream infection (NBSI), congenital microcephaly, LBW, preterm birth, SGA, and stillbirth. Identified cases of each outcome from March 1, 2020 and August 31, 2020 were tabulated at initial case screening, and then refined and subcategorized according to level of diagnostic certainty using GAIA case definitions [22–27]. Additionally, for all identified cases of adverse birth outcomes, maternal antenatal vaccination history was assessed. Lastly, documentation of any birth complications as well as the delivery type were evaluated for all pregnancies.

The initial screening processes as well as the application of GAIA case definitions for the establishment of diagnostic certainty are explained elsewhere [6]. Briefly, instances of stillbirth and NBSI were screened by indication as such in the birth record. Cases of LBW were identified in the screening module according to the World Health Organization (WHO) cutoff of 2500 g. Similarly, preterm births were identified by either indication as such in the birth record or a gestational age at birth recorded as less than 37 weeks in the birth record. To the best of our knowledge, no country-specific pediatric growth charts exist in DRC; thus, cases of congenital microcephaly and SGA were classified using reference charts from the INTERGROWTH-21st project [28] as well as methodology laid out by The Brighton Collaboration Congenital Microcephaly Working Group and the WHO [25, 29]. Each identified case was classified according to GAIA case definition criteria, where Level 1 represents the highest diagnostic specificity and Level 4 represents poor/insufficient information for case confirmation. We additionally included a new category, Level 5, to describe cases that did not meet any diagnostic certainty standards.

The presence of maternal antenatal vaccination for tetanus was recorded using antenatal care records among the mothers of identified adverse birth outcome cases; GAIA criteria were then applied to classify maternal vaccinations [30]. To assess birthing complications, abstractors reviewed archival birth records for the presence of any of the following delivery-related complications: antepartum/postpartum hemorrhage, retained placenta, obstructed labor, maternal death, and other complications. For delivery type, abstractors noted whether the birth record indicated one of the following types of delivery: vaginal birth, Cesarean section, or unspecified.

### Statistical analysis

For this analysis, we refer to the birth records collected from July 1, 2019 through February 28, 2020 as the pre-COVID-19 period, and the birth records collected from March 1, 2020 through August 31, 2020 as the intra-COVID-19 period. Basic demographic information on the mothers and their newborns was tabulated for the intra-COVID-19 period; means with standard deviations (SDs) and medians with ranges were calculated for continuous variables while proportions were provided for categorical variables. Moreover, in the intra-COVID-19 period, the following three sites were further designated as COVID-19 care facilities due to the fact that these facilities had specific areas and/or units for COVID-19 patients: Saint Joseph hospital, Kinshasa general hospital, and Ngaliema clinic.

The prevalence of each birth outcome of interest was estimated in both periods. Birth outcomes of interest were assessed for both intra-site prevalence and an overall prevalence within each period. The overall prevalence of each outcome was compared across time periods via two-sample *z*-test for equality of proportions; statistical significance was determined at the 0.05 level of significance. Additionally, the intra-site prevalence and GAIA classification schemes were compared across time periods via direct comparison. Only descriptive statistics (i.e., frequencies and percentages) were generated for birth complications and birthing type in both time periods.

Furthermore, a sensitivity analysis was performed to determine whether results differed when the date of the first documented case of COVID-19 in DRC (i.e., March 10, 2020) was used as a cutoff between pre-COVID-19 and intra-COVID-19 periods in place of February 28, 2020 [7]. Lastly, a sensitivity sub-analysis was performed among the three health facilities designated as COVID-19 care facilities for LBW, preterm birth, stillbirth, and SGA to better isolate any impacts of the COVID-19 pandemic on these outcomes by comparing across time periods via two-sample *z*-test for equality of proportions. NBSI and microcephaly were not included as outcomes in this sub-analysis due to potential measurement error that we have discussed previously for these outcomes [6]. Statistical analyses were performed using R version 4.0.4 (R Foundation for Statistical Computing, Vienna, Austria).

## Results

### Study population

A total of 14,300 birth records were abstracted across the two study time periods. Records from 7,697 births taking place during the pre-COVID-19 period have been extensively described elsewhere [6]. For the 6,603 birth records extracted from medical archives for review during the intra-COVID-19 period, the number of documented births in the study period ranged from 298 to 943 per delivery center. About one-third (*n *= 2,367) of births recorded in this period met at least one case definition for an adverse event (Table 1). Mothers were on average 28.5 years of age at delivery (SD = 6.36) and had a median of three previous pregnancies (range: 0 to 13). There were more male (53.2%) than female (46.8%) newborns recorded during the intra-COVID-19 period (data not shown). During the intra-COVID-19 period, case type identification from the initial screening module completed for all births in the study period ranged from 1.4% (NBSI) to 18.5% (SGA) of births reviewed (Table 1).

**Table 1:** Screened cases of adverse birth outcomes by study site and overall, March 1, 2020 – August 31, 2020, Kinshasa, DRC

Health Facility	Stillbirth n (%)	Preterm Birth* n (%)	LBW n (%)	SGA n (%)	Microcephaly n (%)	NBSI n (%)	Any Case Type n (%)	Total Records Reviewed n (%)
Bomoi Health Center	4 (0.4)	13 (1.4)	53 (5.6)	55 (5.8)	36 (3.8)	2 (0.2)	92 (9.8)	943 (100.0)
Bondeko Health Center	11 (1.5)	59 (7.8)	86 (11.4)	179 (23.7)	93 (12.3)	34 (4.5)	336 (44.4)	756 (100.0)
Lisanga Health Center	12 (1.4)	69 (8.0)	73 (8.5)	208 (24.2)	10 (1.2)	9 (1.0)	280 (32.6)	860 (100.0)
Siloe Bdom Health Center	13 (2.3)	23 (4.0)	41 (7.2)	38 (6.7)	112 (19.7)	3 (0.5)	157 (27.6)	568 (100.0)
Bosembo Health Center	8 (2.7)	3 (1.0)	49 (16.4)	67 (22.5)	167 (56.0)	1 (0.3)	185 (62.1)	298 (100.0)
Esengo General Hospital	17 (2.7)	23 (3.6)	91 (14.4)	190 (30.0)	62 (9.8)	2 (0.3)	258 (40.7)	634 (100.0)
Mokali General Hospital	19 (4.5)	26 (6.1)	52 (12.2)	34 (8.0)	48 (11.3)	3 (0.7)	119 (27.9)	426 (100.0)
Saint Joseph General Hospital	41 (6.8)	67 (11.1)	82 (13.6)	108 (17.9)	84 (13.9)	23 (3.8)	258 (42.8)	603 (100.0)
Kinshasa General Hospital	97 (15.6)	151 (24.3)	166 (26.7)	127 (20.4)	19 (3.1)	1 (0.2)	338 (54.3)	622 (100.0)
Ngaliema Clinic	9 (1.0)	132 (14.8)	126 (14.1)	213 (23.9)	77 (8.6)	13 (1.5)	344 (38.5)	893 (100.0)
Total	231 (3.5)	566 (8.6)	819 (12.4)	1219 (18.5)	708 (10.7)	91 (1.4)	2367 (35.8)	6603 (100.0)

Row percentages describe site-specific prevalence of each birth outcome of interest. Outcomes are not mutually exclusive

^*^Preterm birth definition met by binary variable indication (yes/no), or by gestational age < 37 weeks recorded in the birth record. (This definition was used in the screening module to determine case module requirement.)

*DRC*  Democratic Republic of Congo*, LBW*  Low birth weight*, SGA*  Small for gestational age*, NBSI*  Neonatal bloodstream infection

### GAIA standards for intra-COVID-19 birth outcomes

**Fig. 1:**
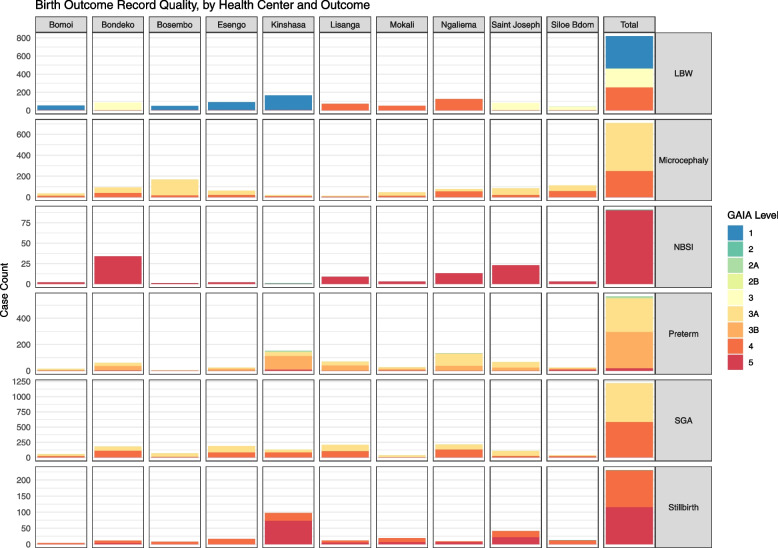
Adverse birth outcomes by study site and overall, according to GAIA classification of diagnostic certainty. GAIA = Global Alignment of Immunization safety Assessment in pregnancy, LBW = Low birth weight, NBSI = Neonatal bloodstream infection, Preterm = Preterm birth, SGA = Small for gestational age

In total during the intra-COVID-19 period, 9.9% of screened cases for any outcome met Level 1 criteria, 0.4% met Level 2, 50.7% met Level 3, and 32.8% met Level 4. The remaining 6.2% of screened cases were categorized as Level 5. All cases of screened LBW met some level of GAIA standard, with almost half categorized at Level 1 (43.8%), and the remaining cases falling into Levels 3 (25.5%) or 4 (30.6%). Virtually all birth records screening positive for preterm birth (96.8%) were classifiable according to GAIA standards; among these classifiable cases, 48.9% met Level 3B criteria, 45.6% met Level 3A criteria, and 2.3% (*n* = 13) met Level 2A criteria. Half of all stillbirth cases identified in the initial screening module were also classifiable by GAIA standards (50.2%). Nearly all of these were classified at Level 4, except for one case from Siloe Bdom health center that was identified at Level 2. Though few cases screened positive for NBSI, only one case from Kinshasa general hospital met any level of diagnostic certainty (i.e., Level 2); thus, the remaining cases were categorized at Level 5. Of screened SGA cases, a majority were categorized at Level 3A (52.4%) with remaining cases falling into Level 4 (47.6%). About two-thirds (65.0%) of microcephaly cases were classifiable at Level 3A, with almost all remaining cases at Level 4; only one screened microcephaly case was unclassifiable and thus labeled as Level 5 (Fig. 1).

### Maternal vaccination during the intra-COVID-19 period

Among the 2,367 mothers of infants identified as a case in the initial screening module during the intra-COVID-19 period, all mothers with recovered tetanus vaccination history for the index pregnancy (*n* = 761, 32.2% of all case mothers) had sufficient information recovered to be classifiable per GAIA standards (Table 2). In total, 399 maternal tetanus vaccinations (52.4% of vaccinated case mothers) were classifiable at GAIA Level 3, and 362 vaccinations were classifiable at Level 2 (47.6% of vaccinated case mothers). No maternal vaccinations were classifiable at GAIA Level 1 due to the fact that there was no evidence of any of the following items identified in the birth record: vaccine name, vaccine manufacturer, and/or vaccine lot number. GAIA comparisons between time periods were not made due to the fact that a large number of maternal vaccinations among mother–child pairs recorded during the intra-COVID-19 actually occurred during the pre-COVID-19 period.

**Table 2:** Maternal tetanus vaccination during index pregnancy for mothers of cases, classifiable by GAIA criteria, by study site and overall

Health Facility	GAIA Definition Met			Level 5 (Missing)* n (%)	Total n (%)
	**Level 1** **n (%)**	**Level 2** **n (%)**	**Level 3** **n (%)**		
Bomoi Health Center	0 (0.0)	67 (72.8)	0 (0.0)	25 (27.2)	92 (100.0)
Bondeko Health Center	0 (0.0)	1 (0.3)	247 (73.5)	88 (26.2)	336 (100.0)
Lisanga Health Center	0 (0.0)	157 (56.1)	3 (1.1)	120 (42.9)	280 (100.0)
Siloe Bdom Health Center	0 (0.0)	0 (0.0)	54 (34.4)	103 (65.6)	157 (100.0)
Bosembo Health Center	0 (0.0)	3 (1.6)	9 (4.9)	173 (93.5)	185 (100.0)
Esengo General Hospital	0 (0.0)	0 (0.0)	0 (0.0)	258 (100.0)	258 (100.0)
Mokali General Hospital	0 (0.0)	33 (27.7)	0 (0.0)	86 (72.3)	119 (100.0)
Saint Joseph General Hospital	0 (0.0)	0 (0.0)	70 (27.1)	188 (72.9)	258 (100.0)
Kinshasa General Hospital	0 (0.0)	0 (0.0)	4 (1.2)	334 (98.8)	338 (100.0)
Ngaliema Clinic	0 (0.0)	101 (29.4)	12 (3.5)	231 (67.2)	344 (100.0)
Total	0 (0.0)	362 (15.3)	399 (16.9)	1606 (67.8)	2367 (100.0)

Maternal vaccination assessed only for those births screened as cases in the initial module. All mothers with a discernable record of tetanus vaccination during the specific pregnancy under investigation from birth record screening (*n* = 731) met at least one Level of GAIA diagnostic certainty criteria for maternal vaccination. Row percentages describe site-specific breakdown of GAIA levels met for maternal tetanus vaccination information

^*^Missing indicates missing vaccination history for the specific pregnancy under investigation, even if maternal records were identified and linked to the birth record. This includes case mothers with a history of tetanus vaccination that occurred prior to the pregnancy that resulted in the birth investigated

*GAIA*  Global Alignment of Immunization safety Assessment in pregnancy

### Pre-pandemic and intra-pandemic comparisons

Birth complications were identified among 22.0% and 14.3% of pregnancies in the pre-COVID-19 and intra-COVID-19 periods, respectively. The status of birth complications was unknown in a higher proportion of pregnancies in the pre-COVID-19 period (25.6%) compared to the intra-COVID-19 period (19.9%). No maternal deaths were reported in the pre-COVID-19 period, yet four maternal deaths (0.4% among identified complications) were reported in the intra-COVID-19 period. The proportion of Cesarean sections was virtually the same in both periods (i.e., 14.8% and 15.4% in the pre-COVID-19 and intra-COVID-19 periods, respectively) (Table 3). The intra-COVID-19 prevalence was similar to the pre-COVID-19 prevalence for all birth outcomes across all study sites, other than preterm birth, which appeared significantly lower in the intra-COVID-19 period than during the pre-COVID-19 period (8.6% vs. 11.5%, *p* < 0.0001). Nonetheless, the prevalence of some conditions differed across the two time periods within study sites; for example, the prevalence of LBW decreased from 22.3% in the pre-COVID-19 period to 16.4% in the intra-COVID-19 period at Bosembo health center (Table 4).

**Table 3:** Comparison of birth characteristics between the pre-pandemic and intra-pandemic periods, overall

Birth Characteristic	Pre-COVID-19		Intra-COVID-19	
	**n**	**%**	**n**	**%**
Birth complications				
No	4031	52.4%	4344	65.8%
Unknown	1971	25.6%	1315	19.9%
Yes	1695	22.0%	944	14.3%
Antepartum/postpartum hemorrhage	140	8.3%	111	11.8%
Retained placenta	7	0.4%	7	0.7%
Obstructed labor	23	1.4%	22	2.3%
Maternal death	0	0.0%	4	0.4%
Other/unknown	1525	90.0%	800	84.7%
Delivery type				
Vaginal	6206	80.6%	5474	82.9%
Cesarean	1137	14.8%	1015	15.4%
Unspecified	354	4.6%	114	1.7%

**Table 4:** Comparison of adverse birth outcome prevalence estimates by study site and overall between the pre-pandemic and intra-pandemic periods

Health Facility	Stillbirth		Preterm Birth		LBW		SGA		Microcephaly		NBSI	
	**Pre**	**Intra**	**Pre**	**Intra**	**Pre**	**Intra**	**Pre**	**Intra**	**Pre**	**Intra**	**Pre**	**Intra**
Bomoi Health Center	1.0%	0.4%	1.7%	1.4%	6.0%	5.6%	6.0%	5.8%	4.1%	3.8%	0.1%	0.2%
Bondeko Health Center	0.8%	1.5%	7.7%	7.8%	9.1%	11.4%	22.3%	23.7%	6.6%	12.3%	1.0%	4.5%
Lisanga Health Center	1.6%	1.4%	6.3%	8.0%	5.5%	8.5%	15.5%	24.2%	0.8%	1.3%	0.6%	1.1%
Siloe Bdom Health Center	1.1%	2.3%	4.0%	4.1%	10.5%	7.2%	10.1%	6.7%	4.8%	19.7%	0.2%	0.5%
Bosembo Health Center	0.3%	2.7%	0.3%	1.0%	22.3%	16.4%	27.1%	22.5%	71.1%	56.0%	0.6%	0.3%
Esengo General Hospital	2.5%	2.7%	7.5%	3.6%	14.7%	14.4%	29.4%	30.0%	15.2%	9.8%	0.3%	0.3%
Mokali General Hospital	5.2%	4.5%	33.8%	6.1%	11.2%	12.2%	8.3%	8.0%	14.3%	11.3%	1.4%	0.7%
Saint Joseph General Hospital	6.8%	6.8%	11.8%	11.1%	12.6%	13.6%	16.7%	17.9%	14.1%	13.9%	7.6%	3.8%
Kinshasa General Hospital	16.6%	15.6%	31.4%	24.3%	29.4%	26.7%	21.8%	20.4%	2.9%	3.1%	0.0%	0.2%
Ngaliema Clinic	0.4%	1.0%	13.6%	14.8%	15.0%	14.1%	23.1%	23.9%	5.6%	8.6%	0.5%	1.5%
Total	3.5%	3.5%	11.5%	8.6%	12.9%	12.4%	17.8%	18.5%	10.1%	10.7%	1.3%	1.4%
p-value*	0.9420		< 0.0001		0.4107		0.3348		0.2299		0.7843	

^*^p-value comparisons are only for the total prevalence*, LBW*  Low birth weight*, SGA*  Small for gestational age*, NBSI*  Neonatal bloodstream infection

**Fig. 2:**
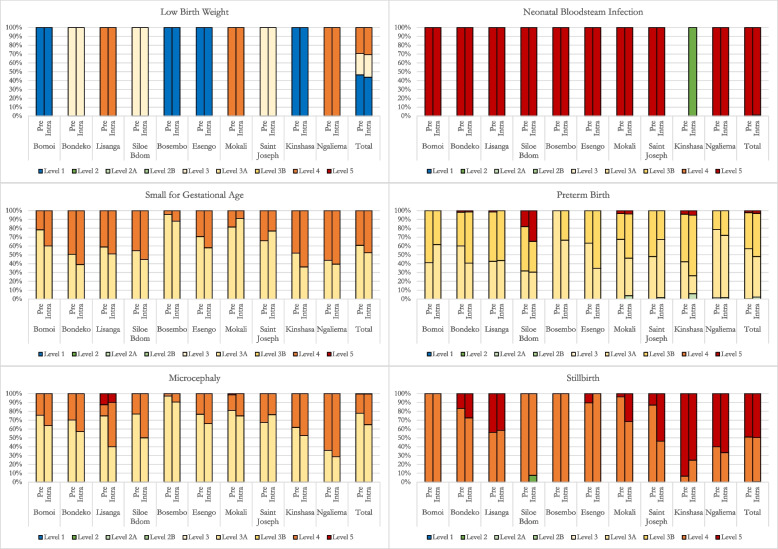
Comparison of diagnostic classification schemes by study site and overall between pre-pandemic and intra-pandemic periods

With regards to GAIA classifications, the level of diagnostic certainty declined slightly from the pre-COVID-19 to the intra-COVID-19 period. Overall, 31.8% of all screened cases were classified as Level 4 or 5 in the pre-COVID-19 period, rising to 39.0% of all screened cases in the intra-COVID-19 period. The proportion of cases classified at Level 2 increased during the intra-COVID-19 period, although the total number of cases classified as Level 2 in either period was very low. This increase in Level 2 classifications was driven primarily by preterm births (specifically at Kinshasa general hospital). At the health facility level, most health facilities followed the general trend of an increase in the proportion of cases classified as Level 4 or 5 in the intra-COVID-19 period (Fig. 2). Classification schemes at a few facilities remained relatively stable for some outcomes. For instance, the classification percentages for every birth outcome except for microcephaly remained generally unchanged at Lisanga health center across time periods. Among the facilities that were designated as COVID-19 care facilities, the proportion of Level 4 classifications slightly increased for microcephaly and SGA from the pre-COVID-19 period to the intra-COVID-19 period (resulting in less Level 3A classifications), except at Saint Joseph hospital. At Saint Joseph hospital specifically, nearly all stillbirths were classified as Level 4 during the pre-COVID-19 period, but only about half were classified as such in the intra-COVID-19 period (with the other half falling into Level 5) (Fig. 2).

### Sensitivity analyses

Sensitivity analyses demonstrated that the results did not significantly change as a result of adjusting the cutoff date for the intra-COVID-19 period (data not shown). For the sensitivity sub-analysis among the three health facilities noted as COVID-19 care facilities, we again did not observe any major differences for LBW, SGA, and stillbirth between the pre-COVID-19 and intra-COVID-19 periods. Of note, a statistically significant drop in preterm births was observed in the intra-COVID-19 period only at Kinshasa general hospital (with no such statistically significant decline observed at either Saint Joseph hospital or Ngaliema clinic). Additionally, the overall GAIA classification schemes appeared mostly consistent between the pre-COVID-19 and intra-COVID-19 periods at all three health facilities (Supplemental Material).

## Discussion

This study examined 14,300 newborn-mother pairs over a 14-month period throughout Kinshasa Province, DRC in order to assess the quality and content of archival medical records and make note of changes in the prevalence of screened maternal and neonatal outcomes in the context of the COVID-19 pandemic. We estimated the prevalence of key adverse birth outcomes using GAIA case definitions and compared these results to data from a birth cohort collected immediately prior to the start of the COVID-19 pandemic officially reaching DRC (i.e., March 2020). We found that most adverse birth outcomes did not significantly decline during the intra-COVID-19 period. Furthermore, we observed that the level of diagnostic certainty of cases recorded in medical archives declined only slightly from the pre-COVID-19 to the intra-COVID-19 periods.

To our knowledge, this study is one of the first investigations into the suitability of GAIA case definitions for use in LMICs during the COVID-19 pandemic. Due to the potentially negative impact of the COVID-19 pandemic on the functionality of health systems [31], it was expected that the overall quality of the data and records keeping would decline, thus leading to less diagnostic certainty for birth and neonatal outcomes of interest. However, our findings suggest that the application and utilization of GAIA case definitions may in fact be durable to disruptive external events (i.e., the pandemic and associated governmental policies) as the overall level of diagnostic certainty fell only slightly across study sites during the intra-COVID-19 period. Although we did observe a drop in the level of diagnostic certainty for some outcomes, particularly microcephaly and SGA, this decline was not severely detrimental across all health facilities (i.e., regardless of whether facilities were designated for COVID-19 care or not). For other outcomes, such as stillbirth and LBW, we did not observe any meaningful drop in the level of diagnostic certainty (at the overall level); for LBW in particular though, it should be noted that case definitions relied entirely on health facility-specific equipment availability and use, which are characteristics that did not change across the time periods. Still, this potential durability is especially salient in a country such as DRC where regular infectious disease outbreaks (e.g., Ebola, cholera, and measles) and associated public health responses are common [32–34]. However, it should be noted that an alternative explanation is that the low number of symptomatic COVID-19 cases recorded in DRC during the intra-COVID-19 period [35] may have not resulted in any meaningful increases in healthcare workload and thus may not have significantly affected records-keeping capabilities at these health facilities. Moreover, it could also be the case that the archival records system already had relatively low diagnostic capability prior to the pandemic (due to missing data and a lack of standardized protocols, for example [6]) that the pandemic did not impact it in any meaningful way.

We also found that the prevalence estimates of microcephaly, LBW, SGA, stillbirth, and NBSI did not change during the intra-COVID-19 period, while the prevalence of preterm birth declined from before to during the COVID-19 pandemic. A clear picture regarding the association between the first wave of the COVID-19 pandemic and adverse birth outcomes has not yet been established in the literature; however, a meta-analysis of forty studies on adverse birth outcomes in seventeen countries concluded that the first wave of the COVID-19 pandemic led to significant increases in the following outcomes only: maternal deaths, stillbirth, ruptured ectopic pregnancies, and maternal depression. Authors of the meta-analysis highlighted that other outcomes showed “considerable disparity between high-resource and low-resource settings.” Of note, Botswana was the only country from sub-Saharan Africa included in that meta-analysis, while high-income countries (such as Italy, the United States of America, and the United Kingdom) were overrepresented [36]. The analysis conducted in Botswana – which was a nationwide birth outcomes surveillance study – reported a 1.27-percentage-point decrease (95% confidence interval: –2.71%, 0.17%) in the risk of preterm birth but no change in the risk of stillbirth during the intra-COVID-19 period [37], a finding mostly in line with the results reported here.

Other observational studies in Argentina [38], China [39], Denmark [40], Iceland [41], Israel [42], and South Korea [43] also reported decreasing preterm birth rates during the intra-COVID-19 period. Although the exact mechanism of this decrease is unknown, some researchers posit that the overall reduction in infectious disease incidence, potential lessening of stress levels due to remote work, and/or the reduction of exposure to air pollution as a result of mitigation measures may all play a role [37, 44]. However, it should be noted that other observational studies (such as those conducted in Austria [45], Canada [46], Jordan [47], and Sweden [48]) concluded that there was in fact no change in the preterm birth rate during the first wave of the COVID-19 pandemic. A similar discrepancy in the literature exists for stillbirth as some investigations (e.g., studies performed in Australia [49], France [50], Spain [51], and Zimbabwe [52]) report the absence of any association between the COVID-19 pandemic and stillbirth, while other investigations (e.g., studies performed in Ethiopia [53, 54], India [55], Italy [56], and Nigeria [57]) reported increased rates of stillbirth during the first wave of the COVID-19 pandemic.

Nonetheless, a more recent study from Naqvi et al. [58] analyzed population-based data on approximately 28,000 births from Maternal and Newborn Health Registry sites in western Kenya, Zambia (i.e., Kafue and Chongwe), and DRC (i.e., North and South Ubangi Provinces) and found that there was no difference in the risk of stillbirth, preterm birth, or LBW during the intra-COVID-19 period compared to the pre-COVID-19 period. Thus, as the mixed results from these studies demonstrate, further research in this area (especially within the context of sub-Saharan Africa) is needed to help better clarify the impact of the COVID-19 pandemic and associated policies on adverse birth outcomes. Regarding birth complications and delivery type though, the aforementioned meta-analysis found that there was no significant change in the Cesarean section prevalence before versus during the pandemic and that this effect was consistent when stratified by high-resource and low-resource settings [36], a finding that aligns with our DRC cohort. For maternal deaths, Chmielewska et al. concluded that the COVID-19 pandemic led to an increase in maternal deaths [36]. Although our study suffers from high levels of missing data for this outcome, our data also tentatively suggests such a potential association between the COVID-19 pandemic and maternal death.

Ultimately, the results of this investigation should be interpreted in light of its limitations. First, it should be emphasized that case identification rates noted here reflect both the true prevalence of the adverse birth and neonatal outcomes of interest in the study population as well as limitations of the information recorded in archived medical records (i.e., measurement error, missing data, etc.). Relatedly, due to the inability of the medical records to provide information on either the full neonatal period (i.e., 28 days) or laboratory findings, the prevalence of NBSI reported here most likely represents an underestimate of the true prevalence; although we attempted to track and link pediatric records to screened birth records for further assessment across the full neonatal period, this process ultimately did not result in a sizable increase in the information needed for NBSI screening/classification. Moreover, crude time-based comparisons conducted here may be subject to uncontrolled confounding (due to differences in care between the two periods, for example). Furthermore, we were not able to account for any potential seasonality of the outcomes due to the crude pre-post method employed here. We therefore recommend that future investigators in this area overcome these limitations by utilizing alternative data (such as the DRC District Health Information System 2 dataset [59]) and/or employing more robust statistical methods that account for confounding, missing data, and potential seasonality.

Additionally, the results of this study may not be generalizable outside of Kinshasa Province; therefore, any conclusions made here do not necessarily apply to other provinces in DRC. Lastly, we were unable to assess the SARS-CoV-2 infection status of mothers included in our cohort; thus, we were only able to make general observations about how the COVID-19 pandemic may have impacted pregnancies and births at the ecological level. These data are therefore not suitable for drawing conclusions about how SARS-CoV-2 infection might physiologically impact pregnancy at the individual level.

## Conclusion

This study succeeded in providing prevalence estimates for key adverse birth outcomes of interest using GAIA criteria during the COVID-19 pandemic. Furthermore, our study adds to the growing literature regarding the impact of the COVID-19 pandemic on maternal and neonatal services and outcomes in Africa and also provides crucial real-world data in the Congolese context. Still, wide discrepancies in prevalence estimates across study sites suggest that measurement error may have impacted medical records. Nonetheless, we hope that this study can help promote the utilization of existing health information in the form of medical records as an innovative way to collect data (as recommended by Nzolo et al. [60]) and thus position such data as an integral part of the surveillance infrastructure within DRC.

## Supplementary Information

**Additional file 1.**

## Data Availability

Data collected and analyzed as a part of this study are available from the corresponding author upon reasonable request.

## Abbreviations

COVID-19: Coronavirus disease 2019
DRC: Democratic Republic of Congo
GAIA: Global Alignment of Immunization Safety Assessment in pregnancy
IRB: Institutional review board
LBW: Low birth weight
LMICs: Low- and middle-income countries
NBSI: Neonatal bloodstream infection
SARS-CoV-2: Severe acute respiratory syndrome coronavirus 2
SD: Standard deviation
SGA: Small for gestational age
WHO: World Health Organization
