# Supplementary material for: Comparison of adverse pregnancy and birth outcomes using archival medical records before and during the first wave of the COVID-19 pandemic in Kinshasa, Democratic Republic of Congo: a facility-based, retrospective cohort study
Source: BMC Pregnancy Childbirth. 2023 Jan 16;23:31. doi: 10.1186/s12884-022-05291-w (PMC9841139; doi:10.1186/s12884-022-05291-w)
Supplement: Supplementary file 1 — Additional file 1. [file 12884_2022_5291_MOESM1_ESM.docx]

**Sensitivity Sub-Analyses for Stillbirth, Preterm Birth, Low Birth Weight, and Small for Gestational Age – filtered to only include the three COVID-19 treatment centers (i.e., Saint Joseph Hospital, Kinshasa General Hospital, & Ngaliema Clinic)**

Supplemental Table 1. Comparison of stillbirth, preterm birth, LBW, and SGA prevalence estimates by study site (i.e., only Saint Joseph, Kinshasa General, & Ngaliema Clinic) between the pre- and intra-pandemic periods

| **Outcomes** | **Health Facilities** | | | | | | | | | | | |
| --- | --- | --- | --- | --- | --- | --- | --- | --- | --- | --- | --- | --- |
|  | **Saint Joseph**  **Hospital** | | | **Kinshasa General Hospital** | | | **Ngaliema Clinic** | | | **Combined** | | |
|  | **Pre**  **(*n*=918)** | **Post**  **(*n*=603)** | ***p*** | **Pre**  **(*n*=717)** | **Post**  **(*n*=622)** | ***p*** | **Pre**  **(*n*=1197)** | **Post**  **(*n*=893)** | ***p*** | **Pre**  **(*n*=2832)** | **Post**  **(*n*=2118)** | ***p*** |
| **Stillbirth** | 6.8% | 6.8% | 0.972 | 16.6% | 15.6% | 0.619 | 0.4% | 1.0% | 0.102 | 6.6% | 6.9% | 0.605 |
| **Preterm Birth** | 11.8% | 11.1% | 0.696 | 31.4% | 24.3% | 0.004 | 13.6% | 14.8% | 0.450 | 17.5% | 16.5% | 0.360 |
| **LBW** | 12.6% | 13.6% | 0.585 | 29.4% | 26.7% | 0.266 | 15.0% | 14.1% | 0.589 | 17.9% | 17.7% | 0.849 |
| **SGA** | 16.7% | 17.9% | 0.529 | 21.8% | 20.4% | 0.549 | 23.1% | 23.9% | 0.671 | 20.7% | 21.2% | 0.671 |

*LBW = Low birth weight, SGA = Small for gestational age.*

Supplemental Figure 1. Stillbirth, preterm birth, LBW, and SGA by study site in the intra-COVID-19 period (i.e., only Saint Joseph hospital, Kinshasa general hospital, & Ngaliema Clinic), according to GAIA classification of diagnostic certainty


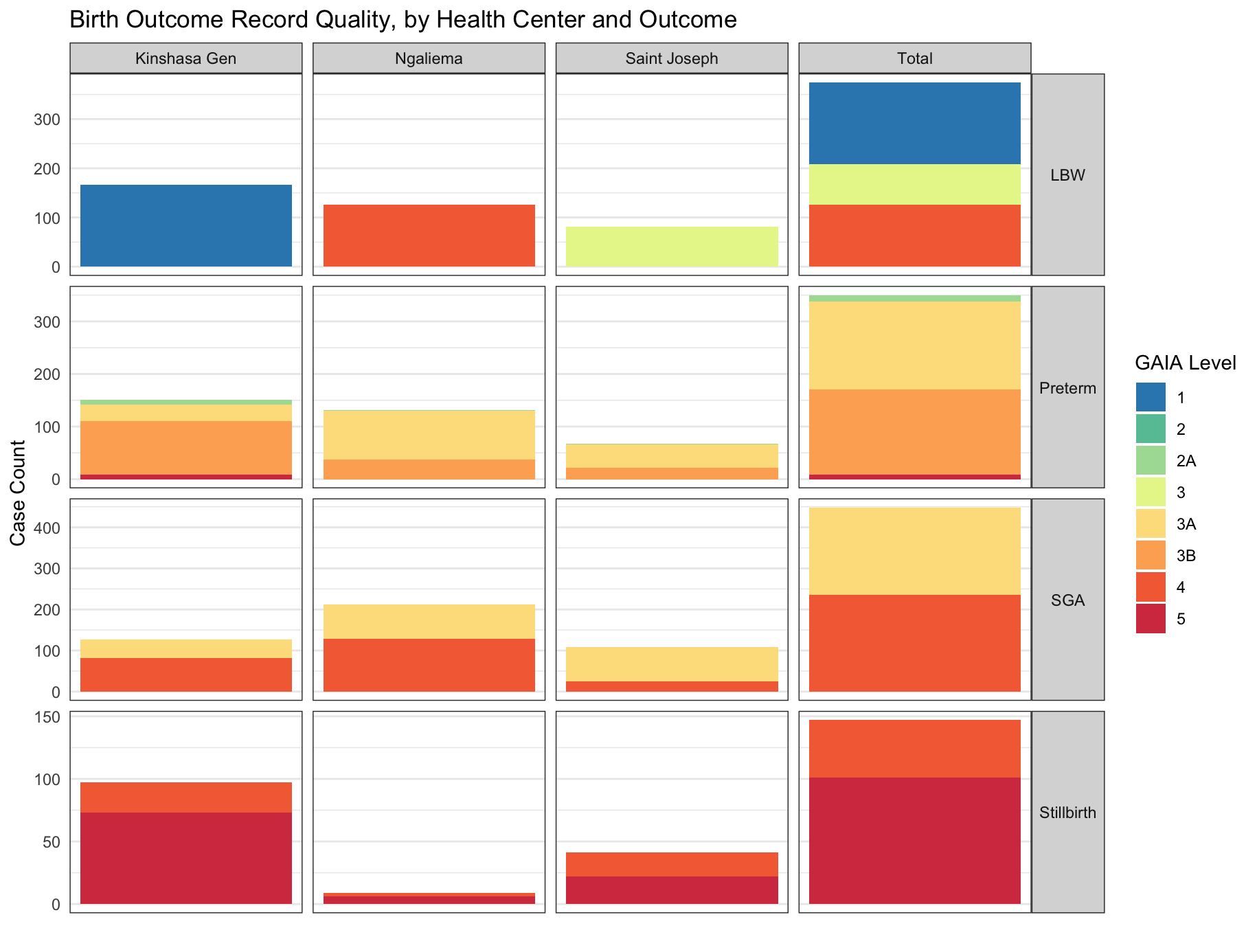


*GAIA= Global Alignment of Immunization safety Assessment in pregnancy, LBW = Low birth weight, NBSI = Neonatal bloodstream infection, Preterm = Preterm birth, SGA = Small for gestational age.*

Supplemental Figure 2. Comparison of GAIA classification schemes for stillbirth, preterm birth, LBW, and SGA by study site (i.e., only Saint Joseph hospital, Kinshasa general hospital, & Ngaliema Clinic) between the pre- and intra-pandemic periods

*GAIA= Global Alignment of Immunization safety Assessment in pregnancy, LBW = Low birth weight, SGA = Small for gestational age.*
